# Thoracoscopic esophagectomy for thoracic esophageal cancer with right aortic arch and Kommerell diverticulum: a case report and literature review

**DOI:** 10.3389/fonc.2023.1215717

**Published:** 2023-09-08

**Authors:** Zhao-Jun Yu, Ling-Wen Guo, Yang-Yun Huang, Lilan Zhao, Zi-Jie He, Xiao-Jie Pan, Wen-Shu Chen

**Affiliations:** Department of Thoracic Surgery, Fujian Provincial Hospital, Shengli Clinical Medical College of Fujian Medical University, Fuzhou, Fujian, China

**Keywords:** esophageal cancer, right aortic arch, Kommerell diverticulum, three-dimensional CT reconstruction, the left recurrent laryngeal nerve

## Abstract

**Background:**

Esophageal carcinoma accompanied by a right aortic arch (RAA) is very rare. When combined with Kommerell diverticulum (KD), a right aortic arch forms a vascular ring encircling both the esophagus and trachea. Due to abnormal anatomy of the upper mediastinum, it is very difficult to dissociate the esophagus and its surrounding tissues, especially the left recurrent laryngeal nerve. Herein, we report a case of successful thoracoscopic esophagectomy in an esophageal cancer patient concurrent with a RAA and KD.

**Case presentation:**

A 62-year-old male patient was diagnosed with esophageal squamous carcinoma in the middle esophagus at clinical stage I (cT1N0M0) according to UICC-TNM classification 8th edition. Further examinations revealed RAA and KD. Based on the three-dimensional CT (3D-CT) reconstruction, a Mckeown esophagectomy via a left thoracoscopic approach in semi-prone position was performed. During the operation, the left recurrent laryngeal nerve was accurately exposed and well protected. Postoperatively, severe complications, including anastomotic leakage and recurrent laryngeal nerve palsy, were not observed. The patient was discharged 12 days after the surgery.

**Conclusion:**

Preoperative 3D-CT reconstruction is useful to clarify the vascular malformation in esophageal cancer patients with RAA, and helpful to formulate a reasonable surgical approach.

## 1 Introduction

Right aortic arch (RAA) is a rare congenital vascular abnormality, which occurs in approximately 0.01% of adults (1). When the RAA is combined with Kommerell diverticulum (KD), a vascular ring can be formed in the upper mediastinum, enclosing the esophagus and trachea. Some patients are admitted to hospital because of dysphagia, dyspnea and recurrent lung infection when the esophagus and trachea are compressed (2).

Esophageal cancer remains the most common malignancy, with the seventh incidence all over the world. Moreover, nearly half of new cases of esophageal cancer were in China (3). Different from Western countries, more than 90% of esophageal cancers in China are squamous cell carcinoma (4). Esophageal cancer with RAA is extremely rare. Due to anatomical abnormalities of the superior mediastinum, esophagectomy is hard to perform. Only a few cases have been reported in previous literatures, and most of surgeries were performed through thoracotomy (5). Herein, we report a case of esophageal cancer concurrent with RAA and KD. Preoperative three-dimensional CT (3D-CT) was reconstructed to clearly demonstrate vascular variation. Furthermore, a thoracoscopic-laparoscopic Mckeown esophagectomy via a left thoracic approach in semi-prone position was successfully performed.

## 2 Case presentation

**Figure 1:**
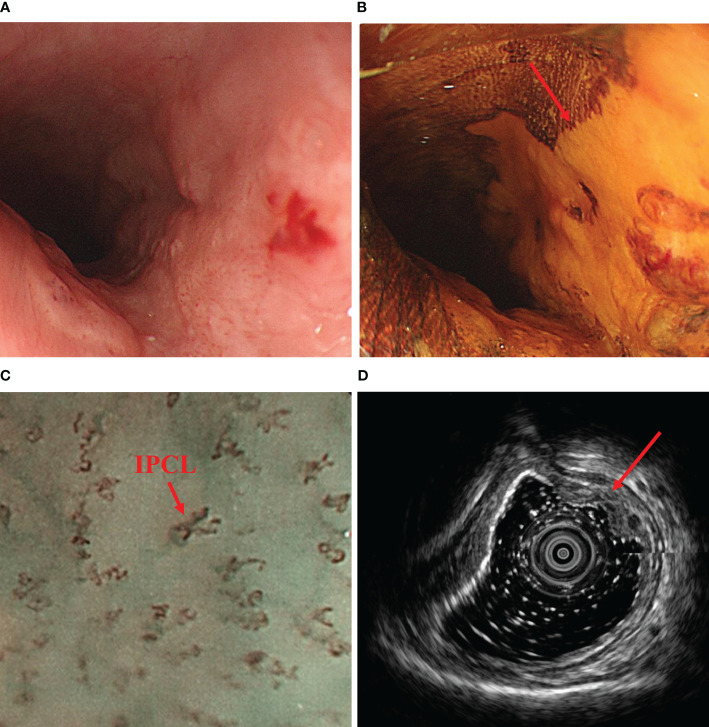
Preoperative endoscopic findings. **(A)** shows a tumor found 25-31 cm from the incisors under upper gastrointestinal endoscopy. **(B)** shows distinct unstained areas in the tumor area under iodine staining (Lugol’s solution, 5% elemental iodine and 10% potassium iodide). **(C)** demonstrates the intraepithelial papillary capillary loop (IPCL) typical of esophageal tumors under magnifying endoscopy (100x). **(D)** shows the tumor invading the submucosa on ultrasound gastroscopy. IPCL, intraepithelial papillary capillary loop.

**Figure 2:**
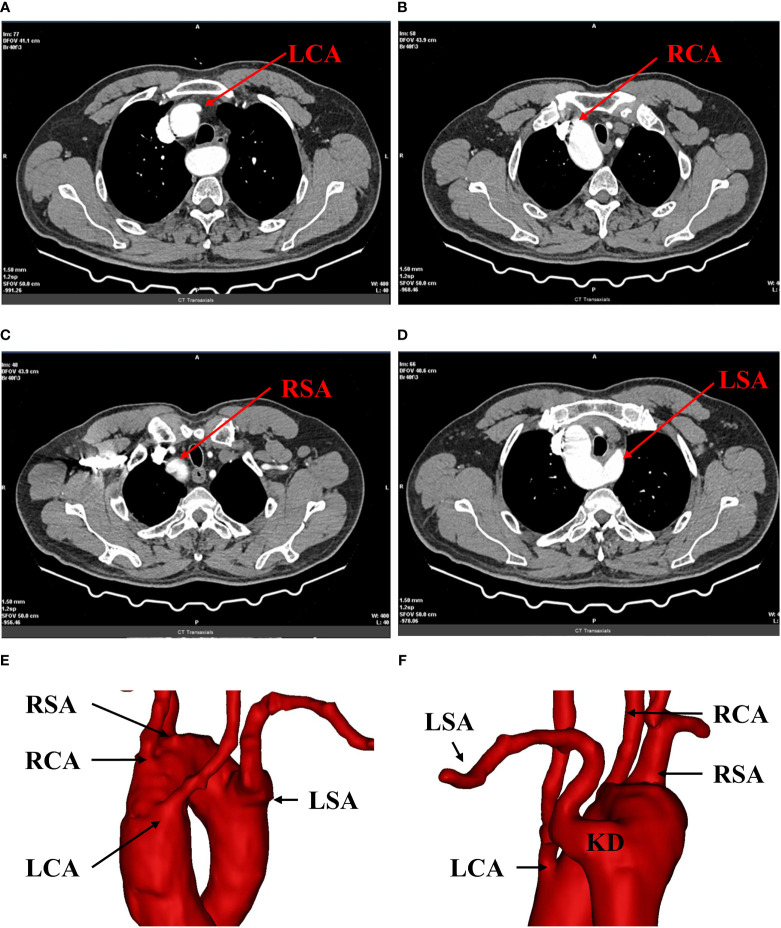
Preoperative 3D-CT reconstruction. **(A)** shows the opening of the left common carotid artery; **(B)** shows the opening of the right common carotid artery; **(C)** shows the opening of the right subclavian artery; **(D)** shows the opening of the left subclavian artery. **(E, F)** show the right aortic arch with branches and KD. LCA, left common carotid artery; RCA, right common carotid artery; RSA, right subclavian artery; LSA, left subclavian artery; RAA, right aortic arch; KD, kommerell diverticulum.

A 62-year-old male patient consulted a local physician with a foreign-body sensation when swallowing. Upper gastrointestinal endoscopy revealed a type-IIa tumor located 25-31 cm from the incisors. Squamous cell carcinoma was confirmed by histopathologic examination of the biopsy samples. Ultrasound gastroscopy indicated that the tumor may invade the submucosa (**Figure 1**). Preoperative contrast-enhanced computed tomography showed minor thickening in the middle thoracic esophageal wall, and no significant lymph node enlargement around esophagus. Laboratory tests and tumor markers showed no significant abnormalities and no distant metastases were detected after completion of other imaging examinations. Therefore, the patient was diagnosed with thoracic esophageal cancer (clinical T1bN0M0, according to UICC 8^th^ edition) with RAA and KD. RAA and KD were also detected (**Figure 2**). 3D-CT reconstruction was performed to clarify this abnormal anatomy. Based on the findings of 3D-CT reconstruction, we discovered that the patient had four arteries coming from the aorta in the following order: left common carotid artery (LCA), right common carotid artery (RCA), right subclavian artery (RSA), and left subclavian artery (LSA). The beginning of the LSA was dilated to form a KD. The patient was categorized as Type II according to Stewart classification of RAA based on the order of aortic arch branching (6).

Esophageal cancer invades the submucosa (T1b) with a 15-30% risk of lymph node metastasis (7–10). Treatment options include radical surgery or a combination of endoscopic submucosal dissection (ESD) and radiotherapy. Following a comprehensive Multi-Disciplinary Treatment (MDT) discussion and consultation with the patient’s family, we ultimately decided to perform radical surgical resection.

### 2.1 Intraoperative finding

**Figure 3:**
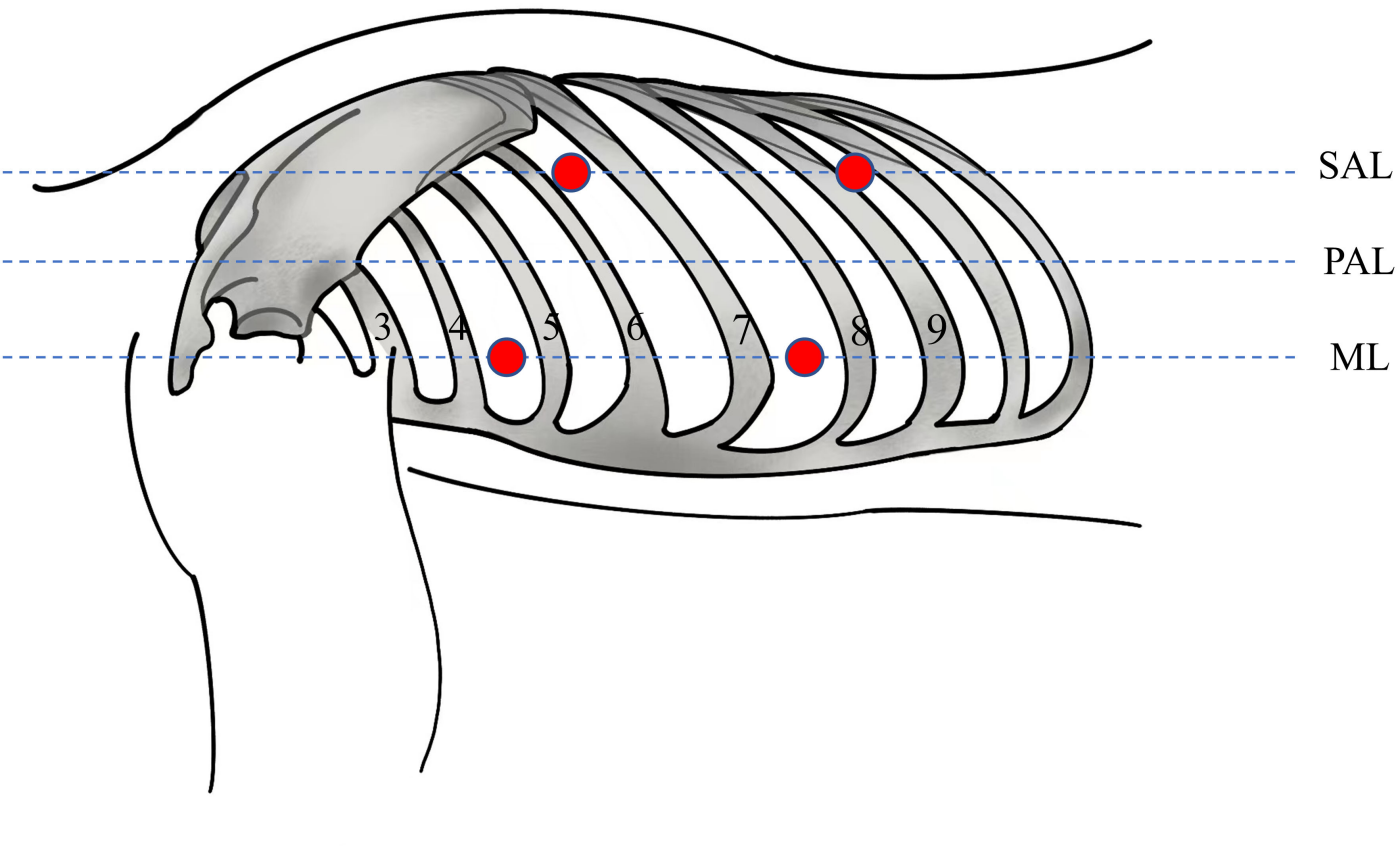
Sites of the four trocars (red circles). The patient assumed a right lateral semi-prone position for the procedure. The main operating port located in the left 4th intercostal mid-axillary line, the observation port in the left 7th intercostal mid-axillary line and the auxiliary operating ports in the 6th intercostal interval in the subscapular angle line and the 9th intercostal interval in the subscapular angle line. ML, midaxillary line; PAL, Posterior Axillary Line; SAL, Subscapular Angle Line.

**Figure 4:**
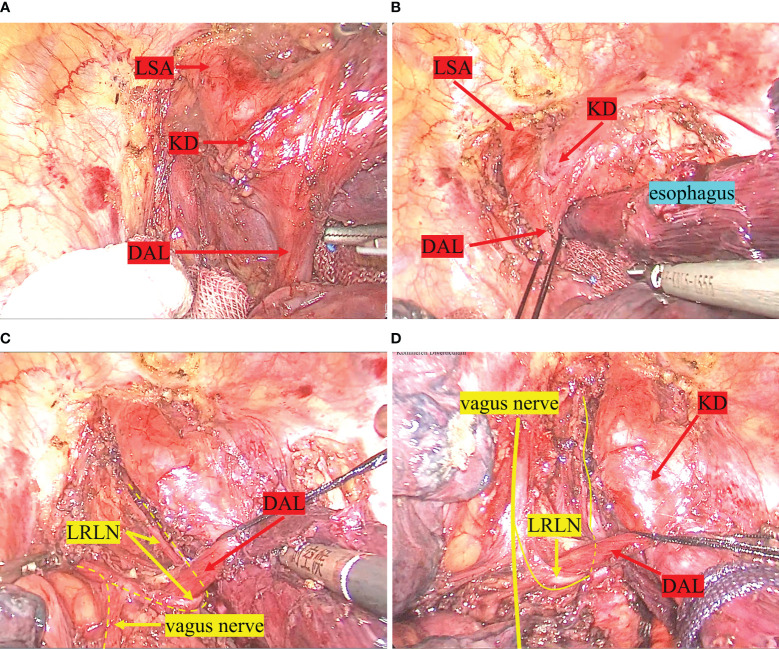
Intraoperative findings. **(A)** shows the ductus arteriosus ligament between the KD and the left pulmonary artery trunk. **(B)** shows the esophagus was surrounded by a ring made up of the ductus arteriosus ligament, the aortic arch, the LSA, and the trachea. **(C)** demonstrates the LRLN emerging from the vagus nerve, bypassing the ductus arteriosus ligament, and traveling up the tracheoesophageal groove. **(D)** shows the upper esophagus being pulled above the ductus arteriosus ligament after the esophagus has been dissected. LSA, left subclavian artery; KD, kommerell diverticulum; DAL, ductus arteriosus ligament; LRLN, left recurrent laryngeal nerve.

A thoraco-laparoscopic esophagectomy was performed for this patient in July 2021. The operation included three steps. Firstly, esophageal dissection and thoracic lymph node dissection were performed via a left thoracoscopic approach in a semi-prone position. The operator and the thoracoscopic assistant are on the ventral side of the patient. The other assistant is on the spinal side of the patient. The thoracoscopic procedure is performed using a 4-port approach with the main operating port located in the left 4th intercostal mid-axillary line, the observation port in the left 7th intercostal mid-axillary line and the auxiliary operating ports in the 6th intercostal interval in the subscapular angle line and the 9th intercostal interval in the subscapular angle line (**Figure 3**). The exposure of the left recurrent laryngeal nerve (LRLN) and the dissociation of the upper mediastinal esophagus are challenging aspects of the operation. During the procedure, we started by the lower and middle esophagus dissociation below the aortic arch and the surrounding lymph node dissection. When we opened the superior mediastinal pleura, we noticed a significant dilation and enlargement of the descending aorta’s initiation. It was determined to be the KD on the basis of preoperative 3D-CT reconstruction findings. Meanwhile, thick, strip-like tissue was observed between the KD and the left pulmonary artery trunk (**Figure 4A**). Based on the results of the preoperative 3D-CT reconstruction, the possibility of a blood vessel was excluded and the ductus arteriosus ligament was considered. The esophagus was surrounded by a ring made up of the ductus arteriosus ligament, the aortic arch, the LSA, and the trachea (**Figure 4B**). The ductus arteriosus ligament was bluntly dissociated and pulled with thick silk thread. We followed the left vagus nerve downwards and located the LRLN behind the ductus arteriosus ligament. We found that it traveled upwards along the groove between the trachea and the esophagus (**Figure 4C**). In the superior mediastinum, the esophagus lies deep within the aortic arch and the LSA. Due to the limited space in the superior mediastinum, it is difficult to dissociate the esophagus. We are also concerned about the possibility of ductus arteriosus ligament damage during the transit of the gastric tube through the esophageal bed during subsequent procedures, which might result in related complications. Therefore, we cut off the esophagus at the beginning of the descending aorta and pulled the upper esophagus out above the ductus arteriosus ligament (**Figure 4D**). We successfully separated the gap between the upper esophagus and the trachea and subclavian artery. This allowed for a complete upper esophageal dissection. Lastly, the upper and lower esophagus stump were secured with sutures.

Secondly, the patient was placed in the supine position, the stomach was dissociated under laparoscopy, and the lymph nodes around the stomach were dissected. Thirdly, a left anterior cervical incision was made to dissociate and resect the cervical esophageal. The gastric tube is pulled through the esophageal bed to the left neck. A mechanical esophageal-gastric anastomosis is performed with a tube anastomosis to restore continuity between the esophageal and the stomach. Among them, the second and third steps were performed in a standard manner without significant differences from conventional surgery.

### 2.2 After surgery

**Figure 5:**
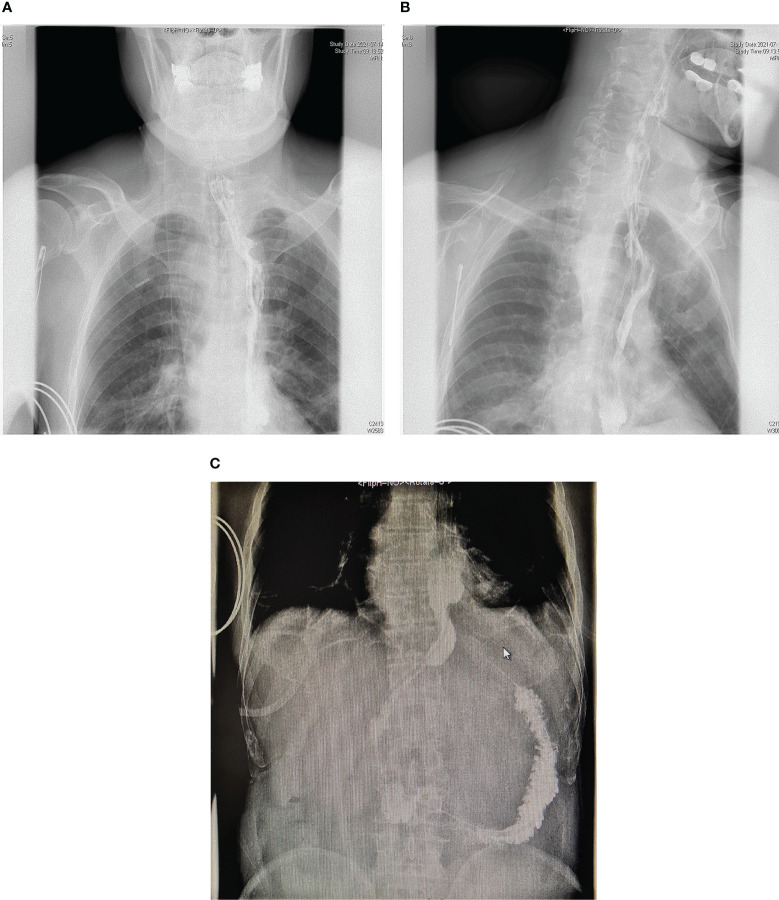
Postoperative upper gastrointestinal imaging. **(A–C)** show the results of the patient’s upper gastrointestinal imaging one week after the operation, with no anastomotic fistula present.

The patient did not experience any complications such as hoarseness due to recurrent laryngeal nerve paralysis or pulmonary infection. Upper gastrointestinal contrast examination was performed one week after surgery, and no anastomotic fistula was found on the radiographic image (**Figure 5**). The patient gradually resumed oral intake and was discharged on the 11th day after surgery. According to the 8th edition of UICC/AJCC staging, the postoperative pathological stage was T1bN0M0 IB.

### 2.3 Follow-up

Follow-up visits were conducted every six months after surgery. The patient has been monitored for two years with no signs of metastasis or recurrence, and is in a favorable state of survival.

## 3 Discussion

**Figure 6:**
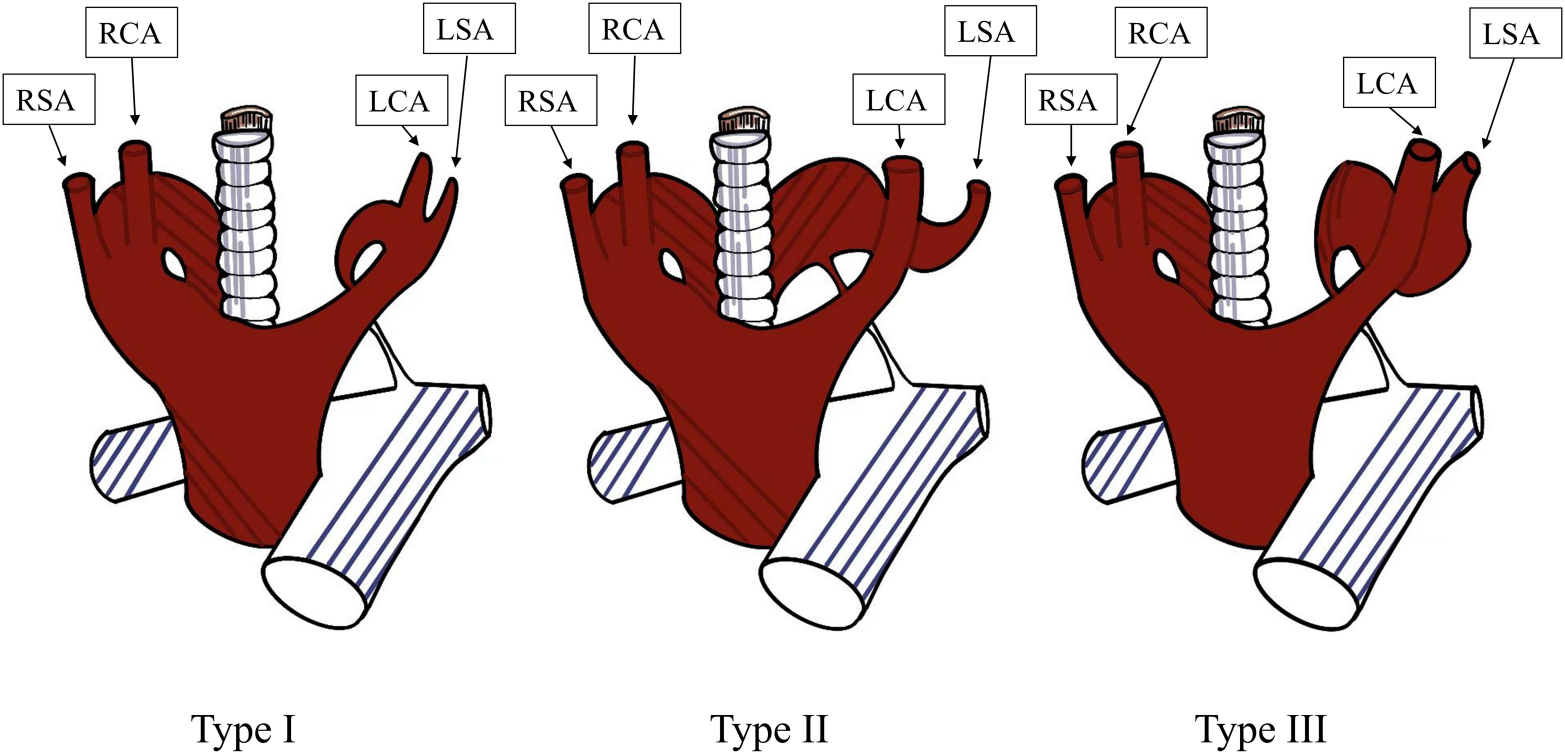
Stewart et al.’s classification of the right aortic arch. Type I is a mirror image of the normal left aortic arch. Type II is characterized by an aberrant LSA. Type III is defined by the following branching order: LCA, RCA, RSA, while the LSA is connected to the left pulmonary artery through the ductus arteriosus. RSA, right subclavian artery; RCA, right common carotid artery; LCA, left common carotid artery; LSA, left subclavian artery.

RAA is a rare congenital cardiovascular anomaly in which the position of the aortic arch is reversed compared to normal individuals, located on the right side of the trachea and esophagus rather than the left side. Although it is usually asymptomatic, it may cause respiratory difficulties, coughing, dysphagia and other symptoms when accompanied by other vascular abnormalities such as double aortic arch, KD, which can form a vascular ring and compress the trachea or esophagus. The incidence of RAA in patients with Tetralogy of Fallot is 13-34%, whereas the incidence in the general population is only 0.01% (11, 12). RAA belongs to Group III of the three types of aortic arch anomalies determined by Edwards based on the development theory of aortic arch (13). Stewart et al. classified the right aortic arch (RAA) into three subtypes based on the order of the branching from the aortic arch (**Figure 6**). Type I is a mirror image of the normal left aortic arch, where the left innominate artery, RCA, and RSA arise sequentially from the ascending aorta. Type II is characterized by an aberrant LSA, where the LCA, RCA, RSA, and LSA arise sequentially from the ascending aorta. Type III is defined by the following branching order: LCA, RCA, RSA, while the LSA is connected to the left pulmonary artery through the ductus arteriosus (6). The case described in this article belongs to the category of type II RAA, in which the aortic arch sequentially gives rise to the LCA, RCA, RSA, and LSA. Moreover, a KD is formed by the LSA’s first segment dilatation.

Esophageal cancer with RAA is extremely rare. The primary challenge in performing surgery for esophageal cancer with upper mediastinal anatomical anomalies is achieving adequate exposure of structures such as the esophagus and blood vessels located in the upper mediastinum. Due to the aortic arch being located on the right side and surrounded by a vascular ring, surgical resection can be quite difficult. Therefore, in the cases reported in the literature, open surgery through the left thorax approach is often used to facilitate exposure and resection of the tumor (5). With the development and maturity of thoracoscopic techniques, thoracoscopic esophagectomy has gradually been applied to the treatment of esophageal cancer with upper mediastinal anatomical anomalies (14). Up to now, including this case, 6 cases have reported thoracoscopic resection of esophageal cancer combined with the RAA (5, 14–17) (**Table 1**).

**Table 1:** Reported cases of thoracoscopic resection of esophageal cancer with RAA.

Authors	Age	Gender	Surgical approach	Surgical methods
N. Kubo et al. (5)	70	Male	left thoracic approach	McKeown
S. Kanaji et al. (14)	52	Male	left thoracic approach	McKeown
J. Linson et al. (17)	73	Male	right thoracic approach	Ivor-Lewis
Y. Ninomiya et al. (15)	70	Male	left thoracic approach	McKeown
S. Nagano. et al. (16)	83	Male	left thoracic approach	McKeown

The key points and challenges during surgery lie in the identification of the arterial duct and the exposure of the LRLN. In the thoracic portion of this case, we performed a thoracoscopic esophagectomy through the left thorax approach. The arterial duct is located between the descending aorta and the main pulmonary artery. It can easily be misinterpreted as a blood vessel. Mishandling can damage vessels at both ends, and even cause fatal bleeding. With preoperative 3D-CT reconstruction, we were able to eliminate out the possibility of the tissue being a blood vessel. In the identification of the recurrent laryngeal nerve, we follow the principle of the retrograde method. First, the left vagus nerve is identified on the anterolateral aspect of the ductus arteriosus. The LRLN is traced inferiorly below the ductus arteriosus. Subsequently, the LRLN loops posterior to the ductus arteriosus and ascends along the tracheoesophageal groove. Due to the narrow gap between the esophageal bed behind the arterial duct, in order to prevent damage to the arterial duct and the LRLN when pulling the esophagus or tubular stomach, we cut off the esophagus at the beginning of the descending aorta, pulled out the upper esophagus above the arterial duct, and restored the continuity of the esophagus using a suture. In this way, when traction is applied to the esophagus or tubular stomach, the LRLN and the arterial duct are kept in a low-tension state to avoid damage.

During surgery, we utilize a semi-prone position and an artificial pneumothorax technique. Due to gravity, the lung tissue collapses towards the abdomen, facilitating exposure of the posterior mediastinum. If resection cannot be performed or bleeding occurs during thoracoscopy, conversion to thoracotomy can be performed immediately. Moreover, artificial pneumothorax contributes to left lung collapse and mediastinal inflation, which results in better exposure of the operative field, especially in the superior mediastinum. For patients with esophageal cancer combined with RAA, trans-hiatal approach or trans-cervical using mediastinal scope esophagectomy can also be performed. The advantage of these approaches is that they can preserve the relative integrity of the thoracic cavity, with minimal interference with vital organs such as the heart and lungs, resulting in faster postoperative recovery (18, 19). However, these approaches have significant limitations. Due to the narrow esophageal bed space above the aortic arch, it is difficult to adequately expose even when inflated. The major difficulty in both trans-hiatal approach and trans-cervical approach using mediastinal scope is that the upper mediastinal space is narrow. In this situation, it is not only easy to injure the relevant tissue but also quite challenging to dissect the lymph nodes (20). The surgical challenge in patients with esophageal cancer and RAA lies in the exposure of upper mediastinal tissues and the dissociation of the esophagus. We performed a combined thoracoscopic and laparoscopic McKeown esophagectomy via a left-sided thoracoscopic approach, which not only gave us ample space to operate and avoid damage to surrounding organs and tissues, but also allowed us to completely clear the lymph nodes in the operative area. Therefore, our method has distinct advantages.

Of course, this method also has some limitations, such as the inability to effectively expose the right recurrent laryngeal nerve and the inability to dissect the lymph nodes along the right recurrent laryngeal nerve (RRLN). In this case, the esophageal cancer was in an early stage, and preoperative imaging evaluation did not reveal local lymph node metastasis. In patients with advanced esophageal cancer, a right thoracoscopy can be added to clear the lymph nodes along the RRLN.

## 4 Conclusion

For patients with esophageal cancer complicated by RAA and KD, esophageal cancer resection surgery can be performed via left thoracoscopy. Preoperative 3D-CT reconstruction is a vital tool for directing surgeons and ensuring safety and efficiency.

## Data availability statement

The original contributions presented in the study are included in the article/supplementary material. Further inquiries can be directed to the corresponding author.

## Ethics statement

The studies involving humans were approved by Ethics Committee of Fujian Provincial Hospital. The studies were conducted in accordance with the local legislation and institutional requirements. The participants provided their written informed consent to participate in this study. Written informed consent was obtained from the individual(s) for the publication of any potentially identifiable images or data included in this article.

## Author contributions

Z-JY and W-SC prepared the manuscript and the literature search. L-WG, Y-YH and LZ collected the clinical and imaging data of the patient. All authors contributed to the article and approved the submitted version.
